# *S100A9* Affects Milk Protein Content by Regulating Amino Acid Transporters and the PI3K-Akt, WNT, and mTOR Signaling Pathways

**DOI:** 10.3390/genes15111486

**Published:** 2024-11-19

**Authors:** Xinyi Zhang, Jun Teng, Zhujun Chen, Changheng Zhao, Li Jiang, Qin Zhang

**Affiliations:** 1 Shandong Provincial Key Laboratory for Livestock Germplasm Innovation & Utilization, College of Animal Science, Shandong Agricultural University, Tai’an 271018, China; gd916@163.com (X.Z.); tengjun0520@163.com (J.T.); chenzhujun155@163.com (Z.C.); chzsdau@163.com (C.Z.); 2 State Key Laboratory of Animal Biotech Breeding, College of Animal Science & Technology, China Agricultural University, Beijing 100193, China

**Keywords:** *S100A9*, milk protein, amino acid transporters, PI3K-Akt, WNT, mTOR signaling pathways, dairy cattle

## Abstract

Background: Calgranulin B (*S100A9*) was found to be strongly associated with milk protein percentage in dairy cattle in our previous genome-wide association study. Methods: SNPs in *S100A9* were identified via pooled sequencing, and genotyping of 1054 cows was performed individually using MassArray with MALDI-TOFMS technology. Association analyses between the *S100A9* SNPs and five milk production traits were conducted using SAS 9.2 software. Functional studies of *S100A9* were conducted using quantitative PCR, Western blot, CCK-8, and immunofluorescence assays. Results: In the present study, we further verified that two SNPs in *S100A9*, g.17115387 C>A and g.17115176 C>A, were significantly associated with milk protein percentage. We found that *S100A9* could affect the expressions of caseins CSN1S1, CSN2, and CSN3 in MAC-T cells by regulating the expressions of amino acid transporter genes. We investigated the effects of *S100A9* on the PI3K-Akt, WNT, and mTOR pathways, which are well known to play important roles in mammary gland development and milk protein synthesis. Our results suggest that *S100A9* regulates the expressions of the relevant genes in these pathways, and thus potentially influences the protein synthesis in the mammary gland. Conclusions: This study demonstrates the important role of the *S100A9* gene in the milk protein trait of dairy cattle and provides new insights into the molecular mechanism of milk protein content.

## 1. Introduction

Milk protein content, along with other milk composition contents including milk fat, lactose, minerals, and vitamins, is one of the main indicators of milk quality of dairy cattle [1]. The main component (~80%) of the milk protein is casein, which is essential for muscle growth, blood pressure regulation, and memory enhancement [1,2,3]. Due to its important economic and nutritive values, the regulation of protein synthesis has always been a research hotspot in the animal genetics field. Studying the molecular mechanism of milk composition synthesis can provide practical benefits for breeding programs of dairy cattle to improve milk quality. In the past decades, considerable efforts have been devoted to identifying the functional genes that affect the milk production traits of dairy cows. So far, 9973 QTLs for milk protein percentage derived from QTL mapping or genome-wide association studies (GWAS) have been reported (QTLdb, https://www.animalgenome.org/cgi-bin/QTLdb/BT/index accessed on 29 October 2023). However, only a few genes have been functionally verified, such as *DGAT1* and *GHR* [4,5].

A former GWAS on milk production traits in a Chinese Holstein cattle population consisting of 6470 cows proved that the *S100A9* gene was strongly associated with milk protein percentages with *p* < 1.0 × 10^−7^ [6]. We also found that *S100A9* was significantly differentially expressed in mammary glands between the early and peak stages of lactation in dairy cows [7]. Given these findings, *S100A9* appears to be a promising functional gene for milk protein traits in dairy cattle. The *S100A9* protein belongs to the S100 family of calcium-binding proteins and is widely distributed in many tissues (including the mammary gland) [8,9]. Previous research demonstrated that *S100A9* was implicated in many physiological processes, such as apoptosis and inflammation, mainly by binding to TLR2, TLR3, TLR4, and RAGE receptors [10,11]. In addition, transcriptome analysis of bovine mammary under heat stress demonstrated that *S100A9* plays a major role in inducing and controlling the inflammatory response, thus affecting the metabolism and milk protein synthesis in the mammary gland of lactating cows [12]. However, there is little information on the regulating mechanism of *S100A9* on milk protein traits.

The present study targeted to achieve the following objectives: (1) Detecting the variants in *S100A9* and validating their associations with milk production traits. (2) Evaluating the effect of *S100A9* on milk protein synthesis in MAC-T cells. (3) Investigating the potential molecular mechanisms of *S100A9*’s effect on milk protein synthesis by analyzing its role in regulating the PI3K-Akt, WNT, and mTOR signaling pathways. It is hoped that our results will provide insights into the potential functions of *S100A9* for milk protein traits.

## 2. Materials and Methods

### 2.1. Ethics Statement

All animal care and treatment procedures were conducted in strict accordance with the Animal Ethics Committee of Shandong Agricultural University, China, and performed in accordance with the Committee’s guidelines and regulations (approval No.: SDUA-2022-112) (12 January 2022).

### 2.2. Detection of SNPs in S100A9

We randomly sampled 50 cows from a population consisting of 1054 Holstein cows from the AustAsia Modern Dairy Farm Co., Ltd. in Shandong Province, China. Blood samples from the 50 cows were collected by a vet from the farm and were stored at −20 °C. DNA was extracted from the blood samples using a blood DNA kit (TIANgel Biotech, Beijing, China) according to the manufacturer’s instructions. Five DNA pools, each comprising ten DNA samples with a DNA concentration of 50 ng/μL, were created by randomly dividing the 50 DNA samples into five groups. Using Primer Premier5.0 (Premier Biosoft, Palo Alto, CA, USA), four pairs of PCR primers were designed based on the cattle *S100A9* sequence (NCBI: NC_037330.1) (Supplementary Table S1). These primers covered the entire coding region and the 2-kb 5′and 3′-flanking regions of *S100A9*. PCR was performed for each pool and the PCR results were sequenced using Applied Biosystems 37306l. SNPs were detected by comparing the sample genomes to the reference genome using Lynnon BioSoft’s DNAMAN application (reference or company). The identified SNPs were individually genotyped using Sequenom MassArray (Bio Miao Biological, Beijing, China) for all the 1054 cows by matrix-assisted laser desorption/ionization time of flight mass spectrometry (MALDI-TOFMS) from Agena Bioscience, San Diego, CA, USA.

### 2.3. Association Analysis for Milk Production Traits

Association analyses between the detected SNPs in *S100A9* and five milk production traits, including milk yield (MY), fat percentage (FP), fat yield (FY), protein percentage (PP), and protein yield (PY) of the first lactation, were conducted in the population of the 1054 cows mentioned above. The daily MY of each cow was obtained from the automatic milking machine on the farm, and FP and PP were determined using the milk component MilkScan FT analyzer (Foss, Hillerod, Denmark) based on MIR technology, and FY and PY were calculated as MY times FP and MY times PP, respectively. We obtained the official estimated breeding values (EBVs) for the five traits for all individuals from the Dairy Cattle Center of Shandong Province, China, and converted the EBVs to de-regressed proofs (DRPs) according to the method described by Garrick et al. [13]. The DRPs were then used as “phenotypic” values for the subsequent association analyses.

For each trait and each SNP, the association analysis was carried out based on the following model:

y=1μ+Zα+e

where **y** is the vector of the DRPs of the trait for all cows, μ is the overall mean, 1 is a vector with all elements of 1, α is effect of the SNP, **Z** is the vector of genotypes (coded as 0, 1, and 2 for the homozygote of the first allele, heterozygote, and homozygote of the second allele, respectively) of the SNP for all cows, and **e** is the vector of random residuals with distribution of $N(0,I\sigma_{e}^{2})$, where I is an identity matrix and $\sigma_{e}^{2}$ is the variance of residuals.

SAS 9.2 software (SAS Institute Inc., Cary, NC, USA) was used to estimate the SNP effect and to test its significance. A *p* value of 0.05 was used as the significance threshold.

### 2.4. Culture of MAC-T Cells and 293T Cells

MAC-T and 293 T cells were cultivated in DMEM media containing 10% fetal bovine serum (Gibco, Grand Island, NY, USA) and 100 units/mL of penicillin and streptomycin. They were maintained at a temperature of 37 °C in a humidified atmosphere containing 5% CO_2_.

### 2.5. Design and Synthesis of siRNAs and Their Transfection in MAC-T Cells

For the *S100A9* knockdown, we designed two silencing siRNAs and a non-silencing siRNA (used as a control) according to the bovine *S100A9* sequence (NM_001046328.2) (Supplementary Table S2). GenePharma Corporation (Shanghai, China) was used to synthesize these siRNAs. A seeding density of 10^6^ cells per plate of MAC-T cells was applied to six-well plates. Following an overnight incubation period in the media, the cells were reconstituted with fresh medium and the siRNAs were transfected at a molar ratio of 1:10 via LipofectamineTM 2000 (Invitrogen, Carlsbad, CA, USA), in accordance with the directions given by the manufacturer. The cells were taken out for RNA extraction after 48 h of incubation.

### 2.6. Construction of S100A9 Over-Expression Vector and Its Transfection in MAC-T Cells

According to the bovine *S100A9* sequence (NM_001046328.2), a set of primers featuring *Xho*I and *Kpn*I restriction enzyme sites was created (forward 5′-GGGGTACCGAGGCTTCTCGGCTTGGTAG and reverse 5′-CCGCTCGAGTTTTTTTGACTCGGAGGAAGAC) using Oligo 6.0 software (https://www.oligo.net/downloads.html accessed on 15 June 2022) for amplifying the ORF of the bovine *S100A9* gene. PCR was conducted using the following protocol: five minutes of initial denaturation at 95 °C, followed by 35 amplification cycles at 95 °C for 30 s, 61 °C for 30 s, and then 72 °C for 1 min. Finally, a last extension was conducted for seven minutes at 72 °C. The PCR product was then purified with the TIANgel Midi Purification Kit (TIANgel Biotech, Beijing, China).

The *Xho*I and *Kpn*I restriction enzymes were utilized to digest the purified PCR product and the pcDNA 3.1(+) eukaryotic expression vector at 37 °C for four hours. Following this, the digested fragments were purified using the Midi Purification Kit (TIANgel Biotech, Beijing, China). The T4 DNA ligase (Takara Biomedical Technologies, Beijing, China) was applied to ligate the *S100A9* cDNA into the pcDNA3.1(+) eukaryotic expression vector. After being amplified in Trans5α chemically competent cells (TransGen Biotech, Beijing, China), the recombinant pcDNA 3.1(+)-*S100A9* was extracted using the EndoFree Maxi Plasmid Kit (TIANGEN Biotech, Beijing, China). Finally, the accuracy of the pcDNA3.1(+)-*S100A9* plasmid sequence was confirmed through restriction enzyme mapping and DNA sequencing analysis.

MAC-T cells were planted in 6-well plates and cultured for a full day. In order to prepare for transfection, the growth media was switched out for one free of antibiotics once the cells had achieved around 80% confluence. The different vectors were introduced into the cells using Lipofectamine™ 2000 (Invitrogen, Carlsbad, CA, USA), following the protocols outlined by the manufacturer. After 48 h of transfection, the cells were collected for detecting the *S100A9* mRNA expression level through quantitative PCR.

### 2.7. RNA Extraction and Real-Time Quantitative PCR

The transfected MAC-T cells were treated with TRIzol reagent (TIANgel Biotech, Beijing, China) to extract total RNA. After quality evaluation of the extracted RNA, each RNA sample in the over-expression and siRNA groups was converted to cDNA by reverse transcription using the Primescript RT reagent kit (Vazyme Biotech, Nanjing, China). Each sample was replicated three times.

Using primers specified in Supplementary Table S3, real-time quantitative PCR (qPCR) was used to assess the expression of *S100A9* and other associated genes. The qPCR was carried out with ChamQ SYBR qPCR Master Mix (Vazyme Biotech, China) on a 96-well plate, with a total reaction volume of 20 µL, utilizing a Roche LightCycler 480^®^ device (Roche, Basel, Switzerland). The *GAPDH* (glyceraldehyde phosphate dehydrogenase) was used as the housekeeping gene for all qPCRs. The quantification of relative mRNA expression levels was determined via the 2^−ΔΔCT^ method [14,15].

### 2.8. Western Blotting

For extracting total cell protein, MAC-T cells were lysed using RIPA lysis buffer (Beyotime, Shanghai, China) with 1 mM PMSF as per the manufacturer’s guidelines. The protein levels were quantified using a BCA protein assay kit from TIANgel Biotech, Beijing. Proteins were put into a PVDF membrane after being electrophoretically separated from protein samples on a 12% SDS-PAGE gel. Next, the membrane was blocked for about two hours at room temperature by 5% skimmed milk (*w*/*v*) [16]. Following overnight incubation at 4 °C along with a primary antibody, it was rinsed with TBST and finally exposed to a secondary antibody for one hour at room temperature. The antibody details and their dilutions are listed in Supplementary Table S4.

### 2.9. Cell Viability Assay

We cultivated MAC-T cells in 96-well plates with a density of 1 × 10^4^ cells per well within an incubator. After incubating overnight in the medium, the cells were transfected with the aforementioned siRNAs via LipofectamineTM 2000 (Invitrogen, Carlsbad, CA, USA) following the guidelines provided by the manufacturer. Cell viability was assessed using the CCK-8 method [17] in accordance with the producer’s recommendations. Briefly, at 12, 24, and 48 h post-transfection, 10 μL CCK-8 was placed into every well, followed by a one-hour incubation at 37 °C in a 5% CO_2_ environment. A microplate reader was used to measure each well’s absorbance at 450 nm.

### 2.10. Immunofluorescence Assay

An immunofluorescence assay was applied to detect the protein-protein interactions. The pCDNA3.1-*S100A9*-HA and pCMV-FLAG-FZD7 plasmids were synthesized by Tsingke Biotech Co., Ltd., Beijing, China. 293T cells were seeded on cell imaging dishes (NEST Biotechnology, Shanghai, China) and co-transfected with the two plasmids. After 48 h of transfection, immunofluorescence detection was performed. Briefly, the cells underwent 30 min of 4% formaldehyde fixation, followed by three washes with PBS and then 35 min of 1% Triton X-100 permeabilization. The blocking step involved incubating the cells in a 10% horse serum solution in PBS at 37 °C for one hour. Afterward, the primary antibodies—rabbit anti-HA and mouse anti-Flag—were introduced and incubated overnight at 4 °C. After giving the cells two PBS washes on the third day, we applied the secondary antibodies tagged with FITC and Cy3 (1:500; FITC-anti-mouse, Abbkine, Wuhan, China; Cy3-anti-rabbit, Beyotime, Shanghai, China) and allowed them to be incubated for two hours at room temperature. We captured the images using a laser confocal microscope.

## 3. Results

### 3.1. Identification of SNPs in S100A9 and Their Associations with Milk Production Traits

After re-sequencing the entire coding region and the 2 kb of the 5′ and 3′-flanking regions of *S100A9* and aligning the sequences to the cattle reference sequence (ARS-UCD1.2) using BLAST, we identified four SNPs, two in the 5′-promoter region (g.17118164 G>A and g.17118494 G>A), one in the third exon (g.17115387 C>A), and one in the 3′UTR region (g.17115176 C>A). By single SNP association analysis, we identified both g.17118164 G>A and g.17118494 G>A was significantly associated with milk protein percentage (*p* = 0.0039 and *p* = 0.0091, respectively) (Table 1), while the remaining two SNPs were not significantly associated with any traits.

### 3.2. Effect of S100A9 on Milk Protein Synthesis in MAC-T Cells

**Figure 1:**
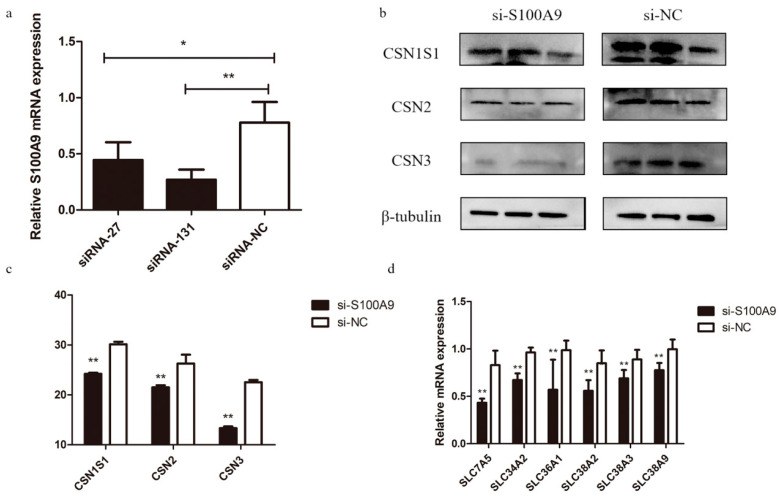
*S100A9* knockdown results in down-regulation of milk casein protein levels in MAC-T cells. (**a**) Expressions of *S100A9* in MAC-T cells transfected with two siRNAs and the non-silencing control (siRNA-NC) revealed by real-time qPCR. (**b**) Western blot analysis of the expressions of three casein proteins (CSN1S1, CSN2, and CSN3) in MAC-T cells transfected with siRNA-131 (si-*S100A9*) and siRNA-NC (si-NC). (**c**) Quantified expression levels of the three casein proteins. (**d**) Expressions of six amino acid transporter genes in MAC-T cells transfected with si-*S100A9* and si-NC revealed by real-time qPCR. Data are presented as mean ± SE. * *p* ≤ 0.05, ** *p* ≤ 0.01 (Student’s *t* test), *n* = 3 for each group.

**Figure 2:**
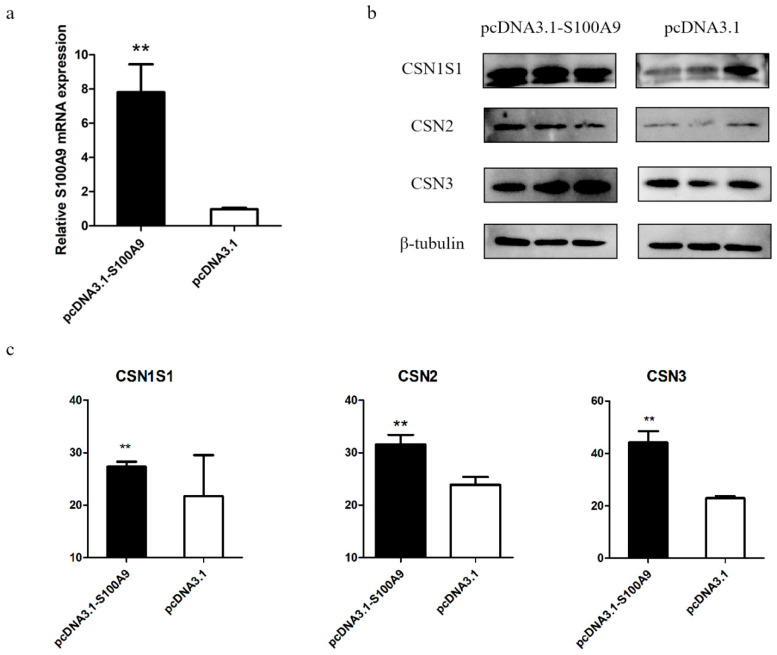
*S100A9* over-expression up-regulates milk casein protein expression levels in MAC-T cells. (**a**) Expressions of *S100A9* in MAC-T cells transfected with overexpression vector pcDNA3.1-S100A9 and control vector pcDNA3.1 revealed by real-time qPCR. (**b**) Western blot analysis of three casein proteins (CSN1S1, CSN2, and CSN3) in MAC-T cells transfected with pcDNA3.1-S100A9 and pcDNA3.1. (**c**) Quantified expression levels of the three casein proteins. Data are presented as mean ± SE. ** *p* ≤ 0.01 (Student’s *t* test), *n* = 3 for each group.

To confirm the effect of *S100A9* on milk protein percentage, we performed *S100A9* RNAi experiment in MAC-T cells. Of the two siRNAs (siRNA-27 and siRNA-131), siRNA-131 showed a stronger silencing effect on the expression of *S100A9* compared to the non-silencing control (si-NC) (Figure 1a). Therefore, siRNA-131 (renamed si-*S100A9* hereafter) was used for the subsequent RNAi experiments. We measured the protein levels of three casein proteins (CSN1S1, CSN2, and CSN3) with Western Blot after *S100A9* silencing and found their expression levels were significantly decreased (Figure 1b,c). We also performed the *S100A9* over-expression experiment in MAC-T cells. The over-expression vector considerably raised the expression of *S100A9*, as shown in Figure 2a, which in turn significantly up-regulated the expression of the three casein proteins (Figure 2b,c). Since the protein synthesis in mammary epithelial cells requires the assistance of amino acid transporters (AATs) on the cell membrane, we also detected the mRNA expression levels of six AAT genes (*SLC34A2*, *SLC38A2*, *SLC38A3*, *SLC38A9*, *SLC7A5*, and *SLC36A1*) and found their expressions were significantly down-regulated after *S100A9* knockdown (Figure 1d), indicating that *S100A9* may affect milk protein synthesis by regulating the expression of AATs.

### 3.3. Effect of S100A9 on the PI3K-Akt Signaling Pathway

**Figure 3:**
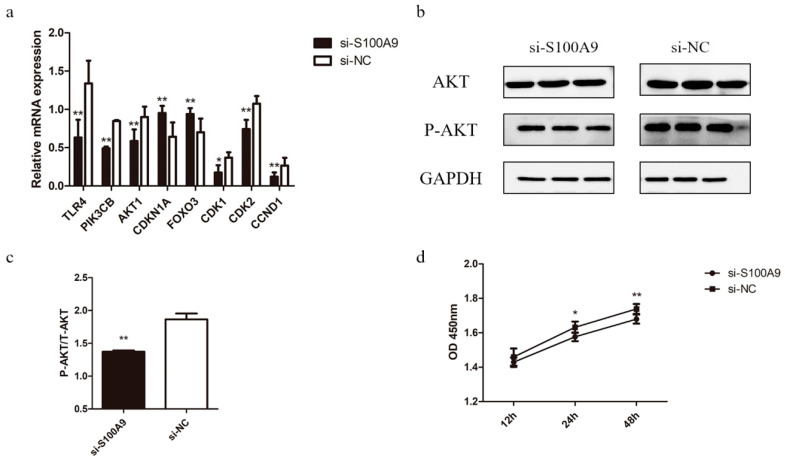
Effect of *S100A9* silencing on genes in the PI3K-Akt signaling pathway. (**a**) Expressions of PI3K-Akt signaling pathway genes in MAC-T cells transfected with si-*S100A9* and si-NC revealed by real-time qPCR. (**b**) Western blot analysis of the expressions of the total AKT protein and phosphorylated-AKT protein (P-AKT). (**c**) Ratio of the phosphorylated to the total AKT protein level. (**d**) Viabilities of MAC-T cells transfected with si-*S100A9* and si-NC for 12 h, 24 h, and 48 h, respectively, revealed by using CCK-8. Data are presented as mean ± SE. * *p* ≤ 0.05, ** *p* ≤ 0.01 (Student’s *t* test), *n* = 3 for each group.

It has been shown that the *S100A9* protein binds to TLR4 to modulate the PI3K-Akt (phosphatidylinositol-3-kinase (PI3K)/serine-threonine kinase (AKT)) signaling pathway [18]. It is well known that the PI3K-Akt signaling pathway is highly related to cellular metabolism, cell growth and proliferation [19]. Therefore, we examined the mRNA expressions of the relevant genes in this pathway after *S100A9* knockdown. In particular, we focused on the downstream genes (*FOXO3*, *CDKN1A*, *CDK1*, *CDK2*, and *CCND1*) in this pathway, which are closely related to cell proliferation [20,21,22]. As shown in Figure 3a, the expressions of *PI3K*, *AKT*, *CDK1*, *CDK2*, and *CCND1* were significantly down-regulated in the si-*S100A9* group, while the expressions of *FOXO3* and *CDKN1A* were significantly up-regulated.

Considering that the PI3K-Akt pathway regulates its downstream genes through phosphorylation [23], we further examined the phosphorylation level of the AKT protein. The findings demonstrated that *S100A9* silencing dramatically decreased the expressions of the total AKT protein as well as the phosphorylated AKT protein (P-AKT) (Figure 3b), while the decreasing extent of P-AKT was much larger than that of the total AKT (Figure 3c). Furthermore, we evaluated the cell viabilities using the CCK-8 method in MAC-T cells at three different time points (12 h, 24 h, and 48 h) after treating the cells with si-*S100A9* and si-NC. As shown in Figure 3d, cell viabilities were decreased significantly for cells treated with si-*S100A9* for 24 h (*p* < 0.05) and for 48 h (*p* < 0.01) compared with those treated with si-NC. These results suggest that the *S100A9* gene may affect the expressions of the downstream genes related to cell proliferation in the PI3K-Akt pathway by altering the AKT protein phosphorylation level.

### 3.4. Effect of S100A9 on the WNT Signaling Pathway

**Figure 4:**
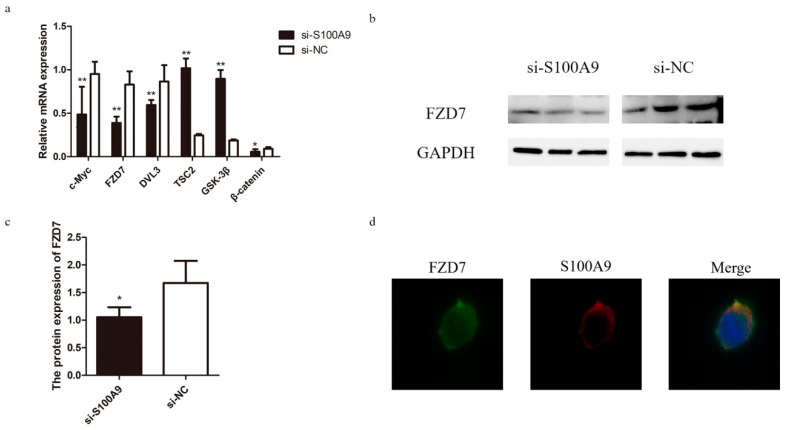
Effect of *S100A9* on genes in the WNT signaling pathway. (**a**) Expressions of the WNT signaling pathway genes in MAC-T cells transfected with si-*S100A9* and si-NC revealed by Real time qPCR. (**b**) Western blot analysis of the protein expression of FZD7. (**c**) Quantified expression level of FZD7. (**d**) Immunofluorescence assay of FZD7 (green) and *S100A9* (red) in membrane of 293T cells. Data are presented as mean ± SE. * *p* ≤ 0.05, ** *p* ≤ 0.01 (Student’s *t* test), *n* = 3 for each group.

*S100A9* has been found to influence the viability and migration of cancer cell lines by regulating the Wnt/β-catenin pathway [24,25]. Some studies showed that this pathway was associated with mammary gland development [26]. These findings are consistent with the results of our MAC-T cell viability experiment mentioned above. Therefore, we investigated the effects of *S100A9* on genes involved in the WNT signaling pathway. The expressions of *β-catenin*, *c-Myc*, *FZD7*, and *DVL3* were significantly down-regulated, while the expressions of *TSC2* and *GSK-3β* were significantly up-regulated after the knockdown of *S100A9* (Figure 4a). We further checked the protein expression levels of the FZD7 protein, which was consistent with the mRNA expression levels of *FZD7* (Figure 4b,c). We then conducted an immunofluorescence assay using 293T cells to explore the interaction between FZD7 and *S100A9*. It turned out that they co-localized on the membrane of 293T cells (Figure 4d), providing evidence for the interaction between the two proteins. The above results suggest that *S100A9* plays a potential regulatory role in the WNT signaling pathway.

### 3.5. Effect of S100A9 on the mTOR Signaling Pathway

**Figure 5:**
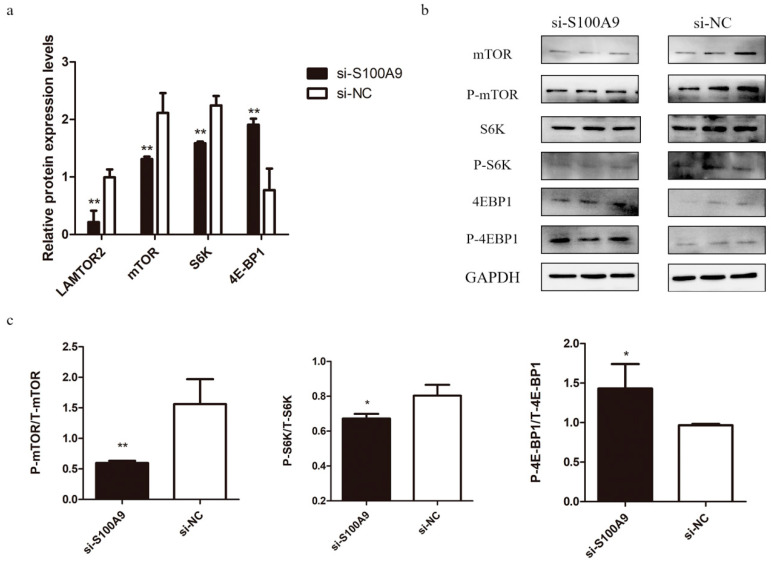
Effect of *S100A9* on the genes in the mTOR signaling pathway. (**a**) Expressions of the genes in MAC-T cells transfected with si-*S100A9* and si-NC revealed by real-time qPCR. (**b**) Western blot analysis of the protein expressions of the total and the phosphorylated mTOR, S6K, and 4E-BP1. (**c**) Ratios of the phosphorylated to the total mTOR, S6K, and 4E-BP1, respectively. Data are presented as mean ± SE. * *p* ≤ 0.05, ** *p* ≤ 0.01 (Student’s *t* test), *n* = 3 for each group.

It is well known that the mTOR signaling pathway plays important roles in the regulation of milk protein synthesis through translation initiation and extension stages [27,28,29]. We examined the effect of *S100A9* on the mRNA expressions of the *LAMTOR2*, *mTOR*, *S6K*, and *4E-BP1* genes in this pathway. The results showed that the expressions of *LAMTOR2*, *mTOR*, and *S6K* were significantly down-regulated, while the expression of *4E-BP1* was significantly up-regulated by silencing *S100A9* (Figure 5a). Moreover, considering the mTOR pathway regulates its downstream genes through phosphorylation [28], we further examined the phosphorylation levels of the mTOR, S6K, and 4E-BP1 proteins. The changes in the total and phosphorylated protein levels of mTOR, S6K, and 4E-BP1 due to silencing *S100A9* were all consistent with that of their mRNA expression levels (Figure 5b,c). These results suggest that *S100A9* has a significant effect on the mTOR signaling pathway.

## 4. Discussion

In this study, we identified two SNPs in the *S100A9* gene, g.17118164G>A and g.17118494G>A, which were associated strongly with milk protein percentage. To reveal the mechanisms of the effect of *S100A9* on milk protein composition, we investigated the regulatory relationship of *S100A9* and the amino acid transporter genes as well as the PI3K-Akt, WNT, and mTOR pathways.

Milk protein is directly synthesized by mammary epithelial cells, utilizing free amino acids absorbed from the bloodstream as its primary raw materials [30]. Since amino acids are polar molecules that cannot freely pass through the cell membrane, they require the assistance of amino acid transporters (AATs) on the cell membrane to enter mammary epithelial cells [31]. Therefore, AATs are essential for protein synthesis in mammary epithelial cells and thus are important for milk protein content. For example, Xing et al. [32] found that overexpression of *SCL7A5*, which encodes L-shaped AAT (LAT1), in mammary epithelial cells of dairy cows led to a notable augmentation in the levels of β-casein in milk. In this study, we found that the *S100A9* knocked-down significantly declined the expressions of six AATs (*SLC34A2*, *SLC38A2*, *SLC38A3*, *SLC38A9*, *SLC7A5*, and *SLC36A1*) (Figure 1d), and correspondingly resulted in significant decreases of the levels of three caseins (CSN1S1, CSN2, and CSN3) (Figure 1b,c), indicating that *S100A9* could regulate milk protein synthesis by affecting the expressions of AATs.

Increasing evidence indicates that the *S100A9* protein could be a new biomarker for inflammation and autoimmune diseases [11,33,34]. It has been reported that *S100A9* binds to TLR4 to initiate downstream signaling pathways such as PI3K-Akt, promoting the secretion of multiple inflammatory factors and amplifying the inflammatory response [18]. PI3K is a lipid signaling kinase that activates 3-phosphoinositol dependent protein kinase-1 (PDK-1) and produces a second messenger 3,4,5-triphosphate inositol (PIP3), thereby activating AKT [16,17]. In the current study, we found that the mRNA expressions of *PI3K*, *TLR4*, and *AKT* were significantly down-regulated along with the *S100A9* knockdown (Figure 3a). The protein phosphorylation level of AKT was also significantly down-regulated due to the *S100A9* knockdown, which induced changes in the expressions of some AKT downstream genes. In particular, the expressions of *FOXO3* and *CDKNIA* were significantly increased, while the expressions of *CDK1*, *CDK2,* and *CCND1* significantly decreased (Figure 3a). These genes are important marker genes related to the cell cycle. Previous studies showed that *FOXO3* and *CDKNIA* are negatively regulated by the PI3K-Akt signaling pathway, and high expression of these proteins leads to cell cycle arrest at the G1/S transition and induces apoptosis [20,21,22]. Indeed, our CCK-8 assay results showed that the MAC-T cells treated with si-*S100A9* exhibited a significant decrease in cell viability (Figure 3d). These findings indicate that *S100A9* regulates the expressions of genes involved in the PI3K-Akt signaling pathway by interacting with TLR4, thereby regulating milk protein synthesis by affecting the proliferation of bovine mammary epithelial cells.

Moreover, some studies have shown that *S100A9* could affect the viability and migration of cancer cell lines through the regulation of the Wnt/β-catenin pathway [24,25]. Our study demonstrated that *S100A9* can bind to the WNT signaling receptor FZD7 located on the cell membrane (Figure 4d). When WNT binds to FZD7, FZD7 acts on the DVL protein in the cytoplasm and inhibits the activity of GSK-3β, blocking the degradation of β-catenin, leading to the β-catenin accumulating in the cytoplasm and entering into the nucleus, ultimately activating the transcription of the downstream target genes (e.g., *c-Myc*, *CCND1*, etc.) [35,36,37]. Our results showed that the silence of *S100A9* decreased the expressions of *FZD7* and *DVL3* in MAC-T cells and resulted in up-regulation of the *GSK-3β* expression and down-regulation of the *β-catenin* expression (Figure 4a). In the meanwhile, the mRNA expressions of *CCND1* and *c-Myc* were also significantly down-regulated (Figure 4a). c-Myc is a transcription factor that plays an important role in the G1 progression of cells [38,39,40]. Eilers et al. [41] reported that the down-regulation of the c-Myc expression led to cell cycle arrest and apoptosis, which is consistent with the results of the cell viability assay in this study (Figure 3d). Therefore, our results suggested that *S100A9* regulates the WNT signaling pathway through binding to FZD7 and ultimately alters the viability of bovine mammary epithelial cells and thus affects milk protein synthesis.

The AATs, PI3K-Akt, and WNT signaling pathways can directly or indirectly lead to the activation of the mTOR signaling pathway. Previous studies have shown that AATs mainly regulate the activation process of mTORC1 [42,43,44]. In particular, SLC36A1 can combine with Rag/GTPase to transmit the signals to the regulator (LAMTOR) to activate mTORC1. On the other hand, the phosphorylated AKT in the PI3K-Akt signaling pathway can directly active mTOR [45,46,47], and GSK-3β in the WNT signaling pathway can regulate TCS2, leading to inhibition of mTORC1 phosphorylation [48]. When the mTOR pathway is activated through the ways mentioned above, the signal is transmitted to its downstream ribosomal protein S6 kinase (S6K) and eukaryotic translation initiation factor 4E binding protein 1 (4E-BP1) [49], which affects the proliferation and differentiation of mammary epithelial cells and milk protein synthesis [45,46,47]. Our results showed that the *S100A9* knockdown induced the down-regulated expressions of *SLC36A1*, *LAMTOR2* (Figure 5a), and *AKT* (Figure 3a), and up-regulated expression of *TSC2* (Figure 4a). These together led to the reduced mRNA and phosphorylated protein levels of mTOR, and correspondingly down-regulation of S6K and up-regulation of the mRNA and phosphorylated protein expression of 4E-BP1. This induces a decrease in the translation of pyrimidine-containing gene mRNAs, which, in turn, affects the synthesis of milk proteins [23]. Thus, these results suggested that *S100A9* may affect milk protein synthesis through directly or indirectly regulating the mTOR signaling pathway.

**Figure 6:**
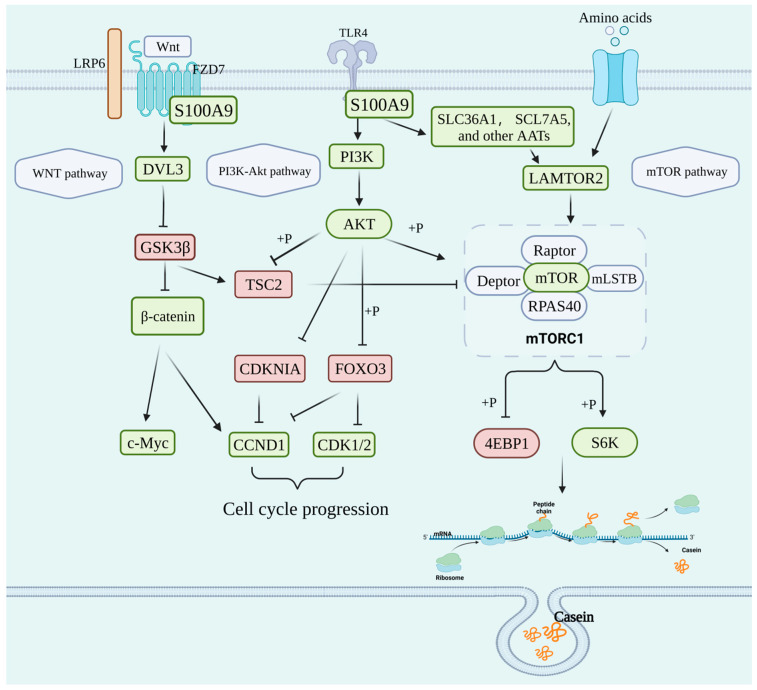
Differentially expressed genes related to mammary epithelial cell proliferation and milk protein synthesis in the PI3K-Akt, WNT, and mTOR signaling pathways after knockdown of *S100A9* in MAC-T cells. Red or green ovals (rectangles) represent the up-regulated or down-regulated proteins (genes), respectively. This figure illustrates the potential underlying mechanisms of the effects of *S100A9* on milk protein content. This figure was created with BioRender.com.

Taking together, we showed that *S100A9* may affect milk protein content by altering the amino acid transporter activities and directly or indirectly regulating some key genes related to mammary epithelial cells proliferation and milk protein synthesis in the PI3K-Akt, WNT, and mTOR signaling pathways, as summarized in Figure 6.

## 5. Conclusions

In conclusion, we identified two SNPs in the *S100A9* gene, g.17115387 C>A and g.17115176 C>A, that are significantly associated with milk protein percentages. We validated that the expression level of *S100A9* could affect the synthesis of caseins (CSN1S1, CSN2, and CSN3) in MAC-T cells. We further demonstrated that *S100A9* regulates protein synthesis by regulating the expressions of the relevant genes in the PI3K-Akt, WNT, and mTOR signaling pathways which are important for mammary gland development and milk protein synthesis. Our findings provide valuable information for elucidating the genetic basis of milk protein metabolism and would contribute to molecular breeding for improving milk protein content in dairy cattle.

## Figures and Tables

**Table 1:** Association analysis between SNPs in *S100A9* and the five milk production traits (data are presented as mean ± SE).

SNP	Mutation	Genotype	N	Milk Yield	Fat Yield	Fat Percentage	Protein Yield	Protein Percentage *
17118164 G>A	G>A	AA	5	44.98 ± 300.55	−15.35 ± 11.77	−0.1331 ± 0.0216	−2.67 ± 8.92	−0.0371 ± 0.0153 ^a^
		AG	141	370.05 ± 47.68	4.24 ± 1.42	−0.0443 ± 0.009	9.77 ± 2.38	−0.0209 ± 0.0045 ^a^
		GG	897	306.71 ± 20.02	2.543 ± 0.61	−0.0316 ± 0.0039	9.15 ± 0.90	−0.0059 ± 0.0018 ^b^
		*p* value		0.3011	0.0973	0.0741	0.5942	0.0039 **
17118494 G>A	G>A	AA	9	19.84 ± 208.043	−11.45 ± 10.37	−0.0915 ± 0.0216	−0.80 ± 6.49	−0.0107 ± 0.0246 ^a^
		AG	127	377.24 ± 48.91	6.68 ± 2.11	−0.0445 ± 0.009	10.00 ± 2.44	−0.0211 ± 0.0046 ^b^
		GG	884	310.09 ± 20.13	4.00 ± 0.94	−0.0319 ± 0.0039	9.25 ± 0.90	−0.0061 ± 0.0018 ^b^
		*p* value		0.1955	0.127	0.2074	0.5846	0.0091 **

* Different superscript letters within a SNP indicate significant differences between means of different genotypes, resulting from the Duncan multiple comparison method. *p* value shows the significance for genetic effects among the SNPs: ** *p* value < 0.01.

## Data Availability

The original contributions presented in the study are included in the article/Supplementary Material, further inquiries can be directed to the corresponding authors.

## Supplementary Materials

The following supporting information can be downloaded at https://www.mdpi.com/article/10.3390/genes15111486/s1, Table S1. Primers and procedures for PCR used in SNP identification. Table S2. The siRNA sequences for the *S100A9*. Table S3. Primer sequences used for quantitative real-time PCR. Table S4. Antibodies used for western blot.
